# A technical note of flex video-assisted anal fistula treatment procedure: Utilizing modified flexible fistuloscope in video-assisted approach for anal fistula laser treatment

**DOI:** 10.1016/j.sopen.2025.03.001

**Published:** 2025-03-07

**Authors:** Okkian Wijaya Kotamto, Tery Nehemia Nugraha Joseph, Clement Dewanto, Natalia Maria Christina, Nadiska Patricia Artha, Marsja Ruthfanny Hutapea, Jeremiah H. Wijaya

**Affiliations:** a Department of Digestive Surgery, Bethsaida Hospital, Tangerang, Banten, Indonesia; b Department of Surgery, RSAB Harapan Kita, Jakarta, Jakarta, Indonesia; c Department of Surgery, Siloam Hospital Lippo Vilage, Tangerang, Banten, Indonesia; d Department of Surgery, Universitas Pelita Harapan, Tangerang, Banten, Indonesia; e School of Public Health and Preventive Medicine, Monash University, Melbourne, Victoria, Australia

**Keywords:** Anal continence, Anal fistula, Minimal invasive, Laser surgery, Vaaft

## Abstract

This study explores an innovative approach for managing complex anal fistulas, known as the flexible video-assisted anal fistula treatment (flex-VAAFT). This technique uses a modified flexible fistuloscope and a laser diode for precise laser ablation. The flexible fistuloscope offers a wider field of view compared to the traditional VAAFT fistuloscope, allowing for better visualization and accurate assessment of the fistula tract's internal anatomy, enabling meticulous debridement and irrigation. We applied the flex-VAAFT approach in seven male patients aged 36 to 66, documenting the external and internal openings, etiology, and fistula type. Seton placement was used in one case, with follow-up periods ranging from 6 to 12 months. Most patients experienced successful healing, with only one recurrence observed. There were no cases of anal incontinence, and the average hospital stay was brief, lasting between 1 and 2 days. The findings suggest that flex-VAAFT is a promising, minimally invasive method for treating anal fistulas, enhancing surgical precision while preserving anal continence.

## Introduction

Perianal fistula is a difficult medical condition defined by the formation of an irregular tunnel or tract between the anorectal canal and the perianal skin. [1] Perianal fistula's prevalence is 1.69 per 10,000 population, affecting people of all ages and genders. [2] Multiple factors, including patient-related risk factors (e.g., patient gender, age, smoking, alcohol, diabetes mellitus, or obesity), might influence the development and outcomes of patients with anal fistula. [3] Perianal fistulas are caused by the establishment of an irregular connection between the anal canal and the perianal skin, which is usually caused by an infection in the anal glands. The infection can cause the formation of a tract, which allows pus and other inflammatory chemicals to drain. The delicate interaction of elements such as anatomical differences, immunological response, and microbial flora adds to the complication of perianal fistula formation. [4] The signs and symptoms of perianal fistulas can be upsetting and have a substantial influence on the quality of life of those who suffer from them. Perianal discharge, typically with pus or blood, and intermittent pain around the anal region are common clinical presentations. During bowel motions, the symptoms may worsen, causing discomfort and more difficulties. Furthermore, edema, redness, and irritation in the perianal area may occur, adding to the total morbidity associated with perianal fistulas.

Therapeutic surgery is essential in the treatment of perianal fistulas. Various surgical treatments are used, ranging from fistulotomy, which opens and drains the fistula tract, advanced flap and seton procedure. The surgical intervention chosen is determined by the fistula's individual characteristics, such as its location, intricacy, and recurrence risk. The goal of surgical care is not only to relieve symptoms but also to promote good healing by minimize the complication by preserve the anal sphincter. [5] One significant factor contributing to recurrence of anal fistula is the incomplete removal of the original tract or unable to recognize its secondary tract, persistence of spasm and hypertonia in the internal anal sphincter post-surgery, inadequate drainage of sepsis, and undetected sepsis during the operation can result in the persistence or recurrence of anal fistulas. Thus, assessing its original and secondary tract become crucial. A pioneering method that provides surgeons with optimal evaluation of fistula tracts is Video-Assisted Anal Fistula Treatment (VAAFT). VAAFT is a minimally invasive surgical technique used in the management of anal fistulas. VAAFT involves the use of a specialized endoscope to visualize the fistula tract, allowing for precise identification of the internal opening and secondary tracts. Introduced by Meinero et al. in 2006, VAAFT emphasizes principles like controlling the internal opening, draining the main and secondary tracts, and preserving anal continence. [6] Recognized for its minimally invasive nature and sphincter-saving benefits, Meinero's VAAFT technique aligns with the goal of preserving anal function while treating complex anal fistulas. By considering these novel techniques in conjunction with established procedures like VAAFT, clinicians can tailor treatment strategies to individual patient needs, potentially improving outcomes and quality of care in anal fistula management.

In this study, we would like to introduce seven cases employing an innovative surgery for the treatment of anal fistula termed the flex-VAAFT approach. The flex-VAAFT approach represents a comprehensive and innovative strategy for the management of complex anal fistulas, akin to the principles of VAAFT. Central to this approach is the utilization of a flexible fistuloscope, a specialized instrument that offers a wider field of view compared to the traditional fistuloscope used in VAAFT. This modification allows for enhanced visualization and precise assessment of the internal anatomy of the fistula tract, facilitating meticulous debridement and thorough irrigation. Compared to Meinero's technique, our approach offers a more flexible, less rigid, and smaller device for anal fistula treatment, providing better device guidance to reach the target and facilitating the definition of secondary tracts. Additionally, the laser utilized in this approach can be adjusted for more precise targeting of the fistula.

By leveraging the advanced capabilities of the flexible fistuloscope, the flex-VAAFT approach aims to optimize outcomes by precise visualization of the fistula tract and provide the suction function of the device while ablation during laser done. The objective of this study is to present a case series detailing the utilization of the flex-VAAFT approach, employing a modified flexible fistuloscope in a video-assisted approach for the treatment of anal fistulas.

## Case series

**Fig. 1:**
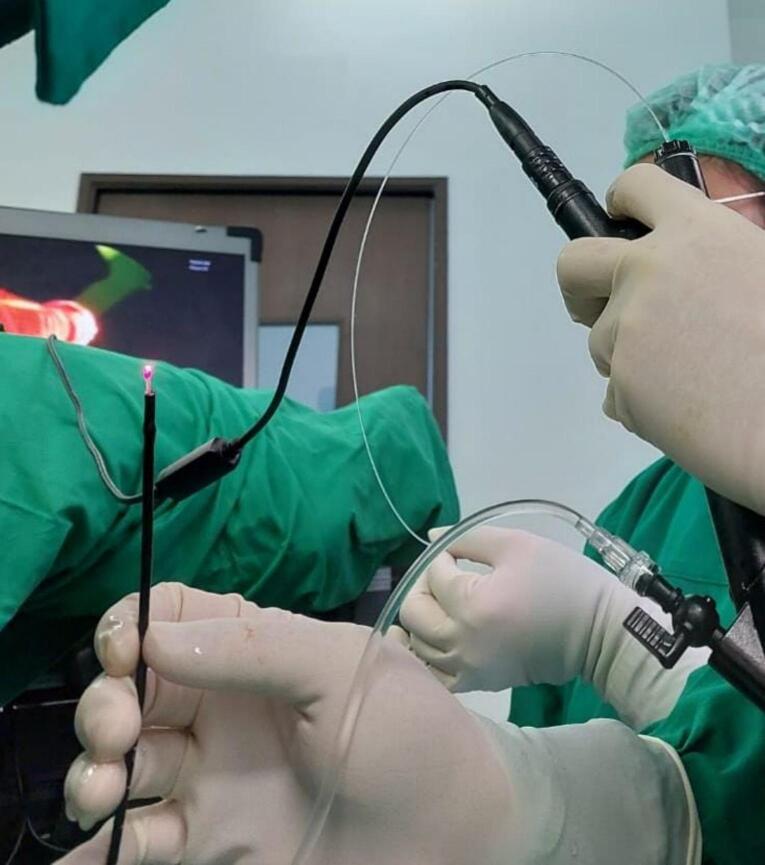
Video ureteroscope used in the flex-VAAFT procedure.

Flex- VAAFT (Fig. 1) in our study used the following surgical apparatus: (1) was employed, utilizing a fistuloscope, (2) video ureteroscope, and (3) 9 Frand a 1470 nm Diode Laser Generator Lasrotronix with a 600um bare fiber laser. The fistuloscope, a compact, flexible video instrument, offered a bending angle of 270 degrees. Its outer diameter during operation was 2.8 mm, with a working channel of 1.2 mm, connected to a monitor. Additionally, necessary equipment comprised 1000 cc normal saline for irrigation, a 270-degree anoscope, small curettage tools, a syringe, betadine solution, cauterization tools, iv canule needles, and ice cubes.

**Fig. 2:**
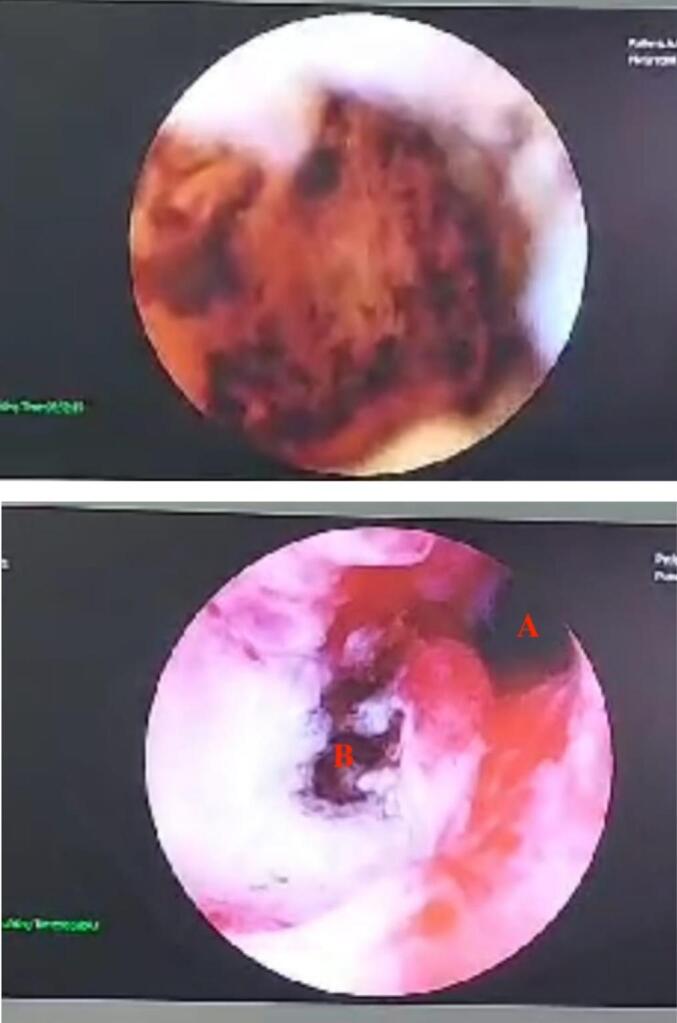
In a case study involving a 46-year-old man, imaging was conducted using the flex-VAAFT, revealing the presence of an anal fistula and associated branches typically not discernible through conventional fistulectomy procedures. A denotes the secondary tract, whereas B the primary anal fistula.

During diagnostic phase, the patient in a lithotomy position under saddle block anesthesia, followed by anal dilatation. Anuscopy facilitated the visualization of the internal opening and other pathological features. Hydrogen peroxide (H₂O₂) was injected into the external opening using a syringe and iv canule, allowing observation of the internal opening through the flow of H₂O₂. To identify secondary tracts and pocket abscesses, a fistuloscope was inserted from the external to internal opening with normal saline irrigation. The identification of fistula branch by flex-VAAFT Flex VAAFT approach is depicted in Fig. 2. The Meinero fistuloscope, used in the VAAFT technique, allows for controlled navigation into the fistula tract with the assistance of an exploratory finger and continuous solution infusion, eliminating the need for an external sheath. The flexible ureteroscope's 270–300-degree bending capability and 2.8 mm diameter enable precise advancement along complex pathways during diagnostic phases, supported by continuous irrigation to distend the tract.

In the operative stage, the fistula tract was curettaged, followed by repeated irrigation using a combination of H₂O₂ and povidone iodine. A fistuloscope was then inserted from the external to internal opening. Once it reached the internal opening, the irrigation tube was replaced with a suction tube to create 20 mmHg negative pressure within the fistula's lumen, clearing remaining debris and fluid while retracting the suction tube. The bare fiber laser was inserted into the working channel and fired with an average power of 12 W from internal to external opening at a speed of 1–2 mm per second. To mitigate the thermal effects of the laser, ice cubes were applied to the skin area around the marked anal fistula. The external opening core was excised using a cauterization tool, followed by debridement of the internal opening.

**Fig. 3:**
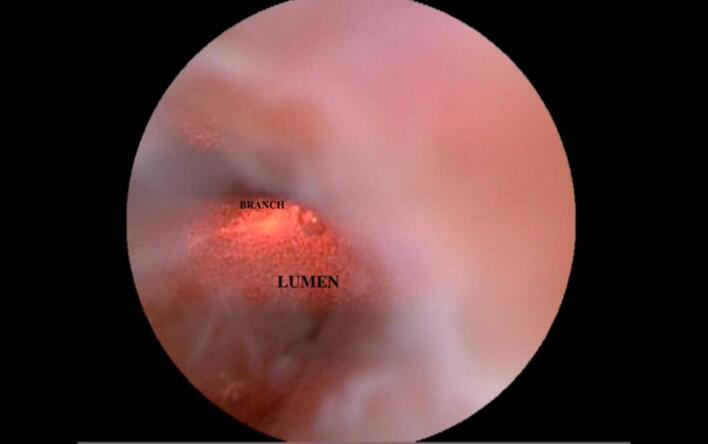
A photograph depicting the treatment of the anal fistula with.

**Fig. 4:**
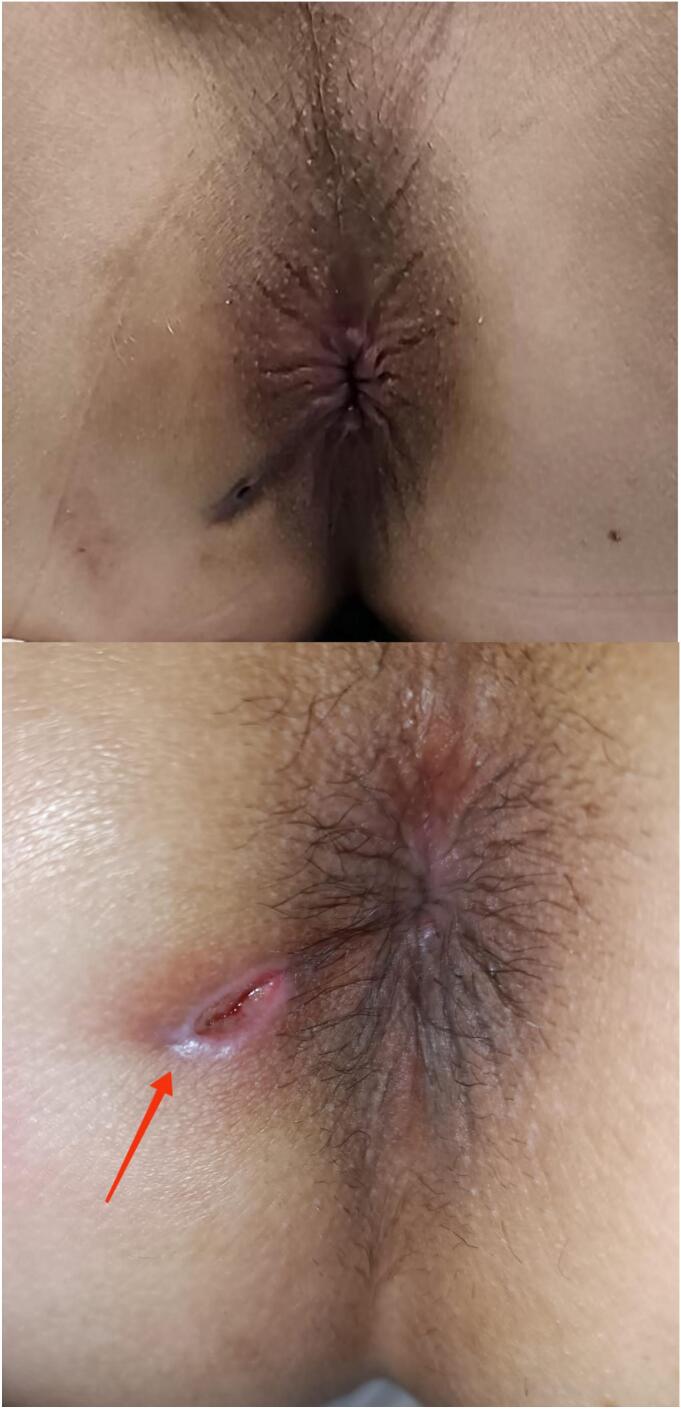
Comparison between pre- and post-operative surgical condition.

This study incorporates modifications to laser techniques for managing anal fistulas, employing a monodirectional laser that facilitates the flow of surgical debris along the irrigation path, with suction at the surgical field's end to remove debris effectively. Laser application follows the diagnostic phase, where irrigation is replaced with suction to create negative pressure, clear debris, and achieve optimal tissue shrinkage temperatures during slow laser retraction. Monodirectional laser fibers were selected due to their compatibility with the 1.2 mm working channel, as radial fibers' larger diameter precludes their use in this scope. While traditional techniques are proficient in managing fistulotomy patients, they lack the ability to identify secondary tracts. Internal orifice management involves debridement with a small curette to reveal healthy tissue, followed by closure with absorbable sutures (Vicryl 0–2) using a continuous interlocking pattern for a water-tight seal. Larger internal openings are managed with an advancement flap to ensure comprehensive treatment of the fistula tract and internal orifice. Additionally, continuous interlocking sutures were applied longitudinally from the rectum using Vicryl 2–0 to secure the closure. Fig. 3, Fig. 4 illustrate the procedural steps in detail.

Healing was evaluated based on the absence of clinical symptoms, including pain, purulent discharge, and swelling, alongside complete wound epithelialization at the external opening site. Recurrence was assessed through monthly follow-ups over a postoperative period of 6 to 12 months, allowing for a thorough and systematic evaluation.

Seven cases of male patients with ages ranging from 36 to 66 years old, all diagnosed with anal fistulas. Almost all af them are reccurent case. The external and internal openings, as well as the etiology and type of fistula, were recorded for each case. Seton was used in one case, and follow-up durations ranged from 6 to 12 months. Most cases resulted in healing, with one case showing recurrence. None of the cases experienced anal continence, and the length of hospital stay ranged from 1 to 2 days. Detailed description of included patients is displayed in Table 1.

**Table 1:** Characteristic baseline for all included 7 patients undergoing flex-VAAFT flex VAAFT procedure.

P	Sex	Age, years	External OP	Internal OP	Etiology	Type	Seton	Fu	Recurrent cases	Result	Anal continence	LOS	VAS
P1	M	60	1 o'clock, 8 cm	12 o'clock,3 cm	Cryptoglandular	Transsphincteric	+	12 m	+	Heal	−	1	1–2
P2	M	45	2 o'clock, 4 cm	2 o'clock, 3 cm	Cryptoglandular	Interssphincteric	−	11 m	−	Heal	−	1	1–2
P3	M	36	7 o'clock, 4 cm	6 o'clock, 3 cm	Cryptoglandular	Transsphincteric	−	10 m	+	Heal	−	1	1–2
P4	M	46	7 o'clock, 3 cm	6 o'clock, 3 cm	Cryptoglandular	Interssphicteric	−	10 m	+	Heal	−	1	1–2
P5	M	66	5 o'clock, 4 cm	6 o'clock, 3 cm	Cryptoglandular	Interssphincteric	−	9 m	+	Residif	−	1	1–2
P6	M	38	8 o'clock, 4 cm	6 o'clock, 3 cm	Cryptoglandular	Interssphincteric	−	6 m	+	Heal	−	1	1–2
P7	M	56	8 o'clock	7 o'clock 15 cm	Cryptoglndular	Transsphincteric	−	10 m	+	Heal	−	1	1–2

## Discussion

This is the first study that describe the flex-VAAFT, a procedure using Flexible fistuloscope to treat anal fistula. Our case series include a total of seven male patients aged between 36 and 66 years old. In one case, a Seton was utilized, and follow-up periods varied between 6 and 12 months. The use of a Seton in anal fistula treatment, within the framework of the Flex VAAFT approach, underscores the methodical and evidence-based strategy employed in managing complex anal fistulas.

Video-assisted anal fistula treatment (VAAFT) has emerged as a minimally invasive technique that focuses on controlling the internal opening, draining the main tract and secondary tracts, and preserving anal continence. VAAFT has shown a wide variation in healing rates for complex anal fistulas, ranging from 22 % to 83.3 % and oftentimes use setons. [2,5] Setons themselves are integral in the management of fistula-in-ano, serving as a crucial intermediary step before definitive procedures such as LIFT. [7] They facilitate the process of downstaging and shortening the fistulous tract, thereby mitigating the likelihood of recurrence. [7,8] This is particularly beneficial in scenarios where a substantial portion of the sphincter muscle is affected or in cases involving complex fistula tracts, as setons aid in resolving sepsis and maintaining continence.

As previously discussed, VAAFT has been recognized for its remarkable ability to maintain anal continence, with the majority of cases exhibiting successful healing and minimal recurrence. Post-procedure incontinence was not reported among patients, highlighting the efficacy of VAAFT in preserving continence function. Understanding anal continence involves recognizing the roles of various muscles, including the internal sphincter, external anal sphincter, and puborectalis muscle, with the puborectal continence reflex playing a crucial regulatory role. [7,9] Factors impacting anal continence, such as changes in anal pressures postoperatively and the role of anorectal sensation, have been explored, with muscle function and reflex mechanisms emphasized as significant contributors to fecal continence. [[10], [11], [12]] In comparison to our procedure, the efficacy of flex-VAAFT approach, coupled with its minimal invasiveness, underscores its value in anal fistula treatment, with the relatively short hospital stays for patients further emphasizing the procedure's swift recovery period. [12] Notably, none of the patients reported anal continence, and their hospital stays ranged from 1 to 2 days.

Laser therapy offers a minimally invasive approach, resulting in faster recovery, decreased discomfort, and shorter hospital stays compared to traditional techniques. [13] Its precision reduces damage to surrounding tissues, making it preferable for fistula excision. In the management of anal fistulas, laser treatment is integral to VAAFT. [13] VAAFT, facilitated by a fistuloscope, allows direct visualization of the fistula tract, enhancing precision and potentially lowering recurrence rates. This method aids in preserving anal continence and provides superior surgical accuracy compared to alternative approaches. Although there is no previous research on the use of a ureteroscope for VAAFT, the ability to identify the internal opening and cauterize the tract under direct vision remains a significant advantage of VAAFT with a fistuloscope.However, in our study, we introduce the flex-VAAFT approach, exemplified in seven cases, as an innovative surgical method for anal fistula treatment. This technique mirrors the principles of VAAFT but employs a flexible fistuloscope, offering a broader field of view for enhanced visualization and precise assessment of fistula tracts. Our modification provides a more flexible and smaller device compared to conventional methods, facilitating better guidance to reach targets and define secondary tracts. Moreover, the adaptable laser used in this approach allows for more accurate targeting of the fistula, thus enhancing treatment outcomes.

However, the mention of one case showing recurrence serves as a reminder of the challenges inherent in managing complex anal fistulas and the importance of ongoing monitoring and adaptation of flex-VAAFT Flex VAAFT approach. Studies have shown varying recurrence rates associated with VAAFT. For instance, a systematic review by Emile et al. reported a weighted mean recurrence rate of 17.7 % for VAAFT with a median follow-up duration of 9 months. [14] On the other hand, Kadhim et al. found a recurrence rate of 26.5 % in their trial. [13] Additionally, a meta-analysis by indicated a fistula recurrence rate of 14.2 % at a median follow-up of 9 months. Within the flex-VAAFT Flex VAAFT approach, such cases may prompt reevaluation of the treatment plan, potentially involving additional interventions or modifications to optimize outcomes and minimize the risk of recurrence.

## Conclusions

In summary, the Flex VAAFT procedure, utilizing an Flexible fistuloscope for anal fistula treatment, demonstrates a methodical and evidence-based strategy, incorporating setons to manage complex cases and emphasizing anal continence preservation. While VAAFT presents another minimally invasive option with varying success rates and also utilizes setons, the Flex VAAFT approach distinguishes itself through its efficacy and minimal invasiveness, leading to shorter hospital stays. However, challenges such as recurrence highlight the necessity for ongoing monitoring and treatment adaptation. Recurrence rates vary across studies, necessitating careful reevaluation of treatment plans to optimize outcomes.

## CRediT authorship contribution statement

**Okkian Wijaya Kotamto:** Writing – review & editing, Writing – original draft, Visualization, Validation, Supervision, Software, Resources, Project administration, Methodology, Investigation, Formal analysis, Data curation, Conceptualization. **Tery Nehemia Nugraha Joseph:** Writing – review & editing, Writing – original draft, Visualization, Validation, Supervision, Software, Resources, Project administration, Methodology, Investigation, Formal analysis, Data curation, Conceptualization. **Clement Dewanto:** Writing – review & editing, Writing – original draft, Visualization, Validation, Supervision, Software, Resources, Project administration, Methodology, Investigation, Formal analysis, Data curation, Conceptualization. **Natalia Maria Christina:** Writing – review & editing, Writing – original draft, Visualization, Validation, Supervision, Software, Resources, Project administration, Methodology, Investigation, Formal analysis, Data curation, Conceptualization. **Nadiska Patricia Artha:** Writing – review & editing, Writing – original draft, Visualization, Validation, Supervision, Software, Resources, Project administration, Methodology, Investigation, Formal analysis, Data curation, Conceptualization. **Marsja Ruthfanny Hutapea:** Writing – review & editing, Writing – original draft, Visualization, Validation, Supervision, Software, Resources, Project administration, Methodology, Investigation, Formal analysis, Data curation, Conceptualization. **Jeremiah H. Wijaya:** Writing – review & editing, Writing – original draft, Visualization, Validation, Supervision, Software, Resources, Project administration, Methodology, Investigation, Formal analysis, Data curation, Conceptualization.

## Ethics approval

We affirm that all subjects within our cohort study have granted informed consent, comprehending the utilization of their data for publication purposes, with strict adherence to confidentiality protocols. Cohort participants have explicitly consented to the publication of their data, with assurances of confidentiality upheld as per the terms outlined during the informed consent process.

## Funding sources

None.

## Declaration of competing interest

All authors declared no conflicting interests.
